# MRI of kidney size matters

**DOI:** 10.1007/s10334-024-01168-5

**Published:** 2024-07-03

**Authors:** Thoralf Niendorf, Thomas Gladytz, Kathleen Cantow, Tobias Klein, Ehsan Tasbihi, Jose Raul Velasquez Vides, Kaixuan Zhao, Jason M. Millward, Sonia Waiczies, Erdmann Seeliger

**Affiliations:** 1 Berlin Ultrahigh Field Facility (B.U.F.F.), Max Delbrück Center for Molecular Medicine in the Helmholtz Association, Robert-Rössle-Str. 10, 13125 Berlin, Germany; 2 Experimental and Clinical Research Center, A Joint Cooperation Between the Charité Medical Faculty and the Max Delbrück Center for Molecular Medicine in the Helmholtz Association, Berlin, Germany; 3 Institute of Translational Physiology, Charité-Universitätsmedizin Berlin, Campus Mitte, Berlin, Germany; 4 Digital Health-Machine Learning Research Group, Hasso Plattner Institute for Digital Engineering, University of Potsdam, Potsdam, Germany; 5 Charité-Universitätsmedizin Berlin, Berlin, Germany; 6 Institute for Medical Engineering, Otto Von Guericke University, Magdeburg, Germany; 7 Guangdong Cardiovascular Institute, Guangdong Provincial People’s Hospital, Guangdong Academy of Medical Sciences, Guangzhou, China; 8 Department of Radiology, Guangdong Provincial People’s Hospital, Guangdong Academy of Medical Sciences, Southern Medical University, Guangzhou, China; 9 Guangdong Provincial Key Laboratory of Artificial Intelligence in Medical Image Analysis and Application, Guangdong Provincial People’s Hospital, Guangdong Academy of Medical Sciences, Guangzhou, China

**Keywords:** Magnetic resonance imaging, Physiology, Kidney, Kidney size, Segmentation, Machine learning

## Abstract

**Objective:**

To highlight progress and opportunities of measuring kidney size with MRI, and to inspire research into resolving the remaining methodological gaps and unanswered questions relating to kidney size assessment.

**Materials and methods:**

This work is not a comprehensive review of the literature but highlights valuable recent developments of MRI of kidney size.

**Results:**

The links between renal (patho)physiology and kidney size are outlined. Common methodological approaches for MRI of kidney size are reviewed. Techniques tailored for renal segmentation and quantification of kidney size are discussed. Frontier applications of kidney size monitoring in preclinical models and human studies are reviewed. Future directions of MRI of kidney size are explored.

**Conclusion:**

MRI of kidney size matters. It will facilitate a growing range of (pre)clinical applications, and provide a springboard for new insights into renal (patho)physiology. As kidney size can be easily obtained from already established renal MRI protocols without the need for additional scans, this measurement should always accompany diagnostic MRI exams. Reconciling global kidney size changes with alterations in the size of specific renal layers is an important topic for further research. Acute kidney size measurements alone cannot distinguish between changes induced by alterations in the blood or the tubular volume fractions—this distinction requires further research into cartography of the renal blood and the tubular volumes.

## Introduction

The kidney is a unique organ. It eliminates water-soluble ‘waste’ products from the body and is pivotal for regulating the balance of sodium, potassium, and water in the body. Maintaining kidney functions is critical for life, and essential for preserving health, especially during aging. The kidneys are comprised of the nephrons, an intricate vasculature, the interstitium, and a capsule that covers the surface of kidney. The nephron is the main functional unit, and is a unique feature of the kidney, responsible for maintaining sodium, potassium, and water balances, and for producing urine. The first structure of the nephron is the glomerulus, where primary urine is formed by ultrafiltration of blood plasma. The glomerulus is followed by the tubule, where the final urine is formed. The tubule reabsorbs the majority of filtered water and osmolytes by energy- and renal oxygenation-dependent processes, and secretes various substances. The volume of fluid within the lumen of the tubules accounts for a large fraction of the kidney volume [1, 2]. Likewise, the blood in the intrarenal vasculature accounts for a large fraction of the kidney volume, particularly in the renal cortex [3–5]. Changes in both the tubular volume fraction and the blood volume fraction are mirrored by changes in kidney size. Due to the relatively rigid renal capsule composed of fibrous proteins, changes in the tubular volume fraction will also influence the blood volume fraction, and vice versa. In general, changes in kidney size due to alterations in the tubular, vascular, or interstitial volume fractions could indicate pathophysiologic developments. Therefore, kidney size is important, because it is a clear macroscopic indicator with a physiological meaning that can help decipher the associations and determinants of renal disease. The growing number of reports in the literature enthusiastically referring to renal size is a testament to its value to renal (patho)physiology [6, 7].

An assessment of kidney size requires non-invasive imaging that supports longitudinal studies—this is the forté of MRI [8, 9]. MRI is a mainstay of diagnostic imaging, offering exquisite anatomic detail. The soft-tissue contrast mechanisms inherent to MRI allow the kidney to be differentiated from surrounding tissue, and facilitate discrimination of the renal layers [10]. An increasing body of literature describes the potential of MRI for quantifying kidney size as a metric that can improve prediction and interception of renal disease, inform on the stages of renal pathophysiology and disease progression, and evaluate responses to treatment. Monitoring kidney size is also crucial for interpreting and understanding the physiological meaning of MRI findings in the kidney, including those seen in acute pathophysiological scenarios. This has profound implications for nephrology and physiology, and requires the integration of a broad spectrum of imaging sciences, data science, radiology, and other related fields of basic science and clinical research.

Recognizing the progress, caveats, and rich opportunities of renal size quantification, this review is not only intended to underscore the strengths of MRI for kidney size assessment. It is also meant to inspire the MRI and biomedical imaging communities to foster research and to spearhead explorations into solving the remaining methodological gaps and unresolved questions in kidney size assessment. The common goal is to gain new insights into renal pathophysiology at the interface of MRI science, image analysis and visualization, physiology, and medicine. To meet this goal, we first discuss common methodological approaches used for MRI of the kidney and their implications for quantifying renal size. Next, we describe approaches tailored for renal segmentation and size quantification from MRI data, together with recent progress and remaining limitations. Early and frontier applications of MRI of kidney size in experimental models and human studies are outlined, along with their implications for preclinical research and clinical science. A concluding section ventures a glance beyond the horizon and explores future directions of MRI of kidney size. MRI of kidney size is an area of vigorous ongoing research, and many potentially valuable developments will receive only brief mention here.

## Why does renal size matter?

Before delving into MRI pulse sequences and dice coefficients, we must first address renal (patho)physiology to gain a deeper understanding as to why renal size matters. The tubular volume fraction (TVF) comprises a large portion of the total kidney volume, and is altered in many clinical scenarios. It can change due to alterations in (i) glomerular filtration rate (GFR), (ii) tubular water reabsorption, (iii) transmural pressure of the tubules, and (iv) outflow of final urine into the extrarenal urinary tract [2]. Pathophysiologically relevant decreases in the TVF can result from a primary decrease in GFR due to reduced effective filtration pressure in a variety of clinical scenarios, including severe renal artery stenosis, circulatory shock, low arterial target pressure during cardiopulmonary bypass, and surgery involving clamping of the suprarenal aorta or the renal artery [11–16]. Other causes of decreased TVF include decreased hydraulic conductance of the filter, which occurs in some forms of glomerulonephritis, and diffuse tissue fibrosis [15, 17–19]. Increases in TVF due to a primary GFR increase are typical for the early stages of diabetic kidney disease [20]. TVF also increases in scenarios with reduced water reabsorption, such as following therapeutic administration of diuretics (particularly osmotic and loop diuretics), and due to hyperglycemia, and in the remaining kidney following unilateral nephrectomy [20, 21]. Increased tubular pressure resulting from increased tubular fluid viscosity following administration of X-ray contrast media during transcutaneous cardiac procedures also increases TVF [22, 23]. Another cause for increased TVF is obstruction of the extrarenal urinary tract, including the renal calices, pelvis, ureter, bladder, and the urethra. Such obstructions can be caused by congenital malformations, kidney stones, tumours, scar tissue, and hyperplasia of the prostate, and can also occur during endourologic procedures [24–27]. Polycystic kidney disease is also characterized by a progressive increase in the TVF [28].

Perturbations that increase tubular volume usually also increase intratubular pressure and intrarenal pressure. As the renal capsule is rather rigid, significant volume and pressure changes can result in *intrarenal compartment syndrome*, in which intrarenal blood vessels are compressed, causing lowering of renal blood flow and thereby impaired renal oxygen supply [27, 29]. This is detrimental for O_2_-consuming tubular reabsorption and the functional state of the kidney as a whole.

Active renal vasomotion or passive circular vessel distension or compression are causes for alterations in the blood volume fraction (BVF), which typically exceed those for other tissues during various (patho)physiological states of the kidney[3, 30, 31]. Clinical scenarios with increased BVF include obstruction of the renal vein during surgical procedures, but also due to long-term conditions such as renal cell carcinoma-derived thrombus formation [11, 32–37]. All of these can result in intrarenal compartment syndrome, that results in an impairment of oxygen supply. Renal artery stenosis, circulatory shock, and surgery involving clamping of the suprarenal aorta or the renal artery are further clinical scenarios that can result in decreased BVF.

Remodeling of renal tissue structure due to fibrotic alterations primarily affects the extracellular matrix of the interstitial volume fraction [17]. Renal tissue fibrosis may be induced by acute kidney injury. In chronic kidney diseases of various origins, progressive fibrosis leads to atrophy of tubular endothelium and rarefication of blood vessels. In these cases, kidney size becomes progressively reduced [17, 38–40]. Changes in the kidney macromorphology, such as formation of renal cysts, renal masses, or localized renal tumours, may induce a net-increase in the different volume fractions.

## Which MRI approaches fit the needs of kidney size assessment?

**Fig. 1:**
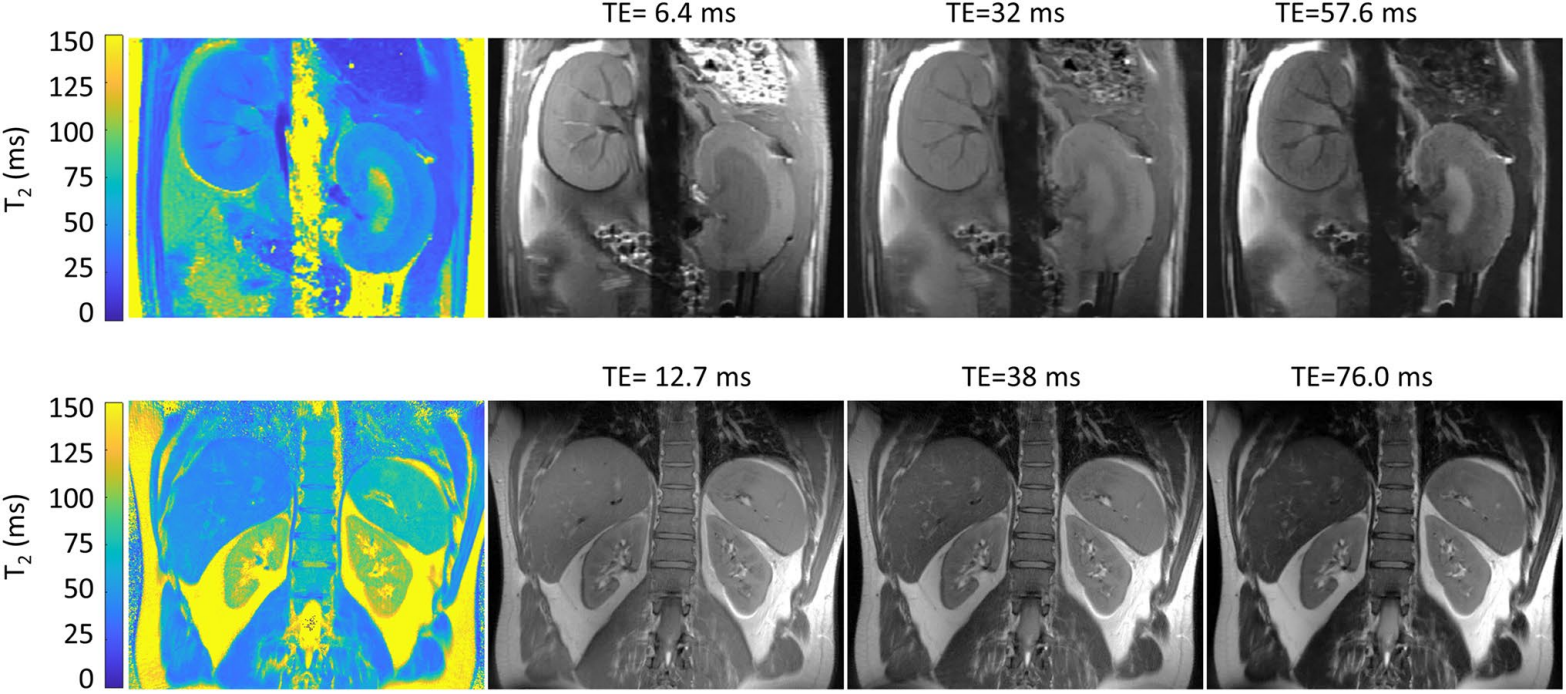
Examples of MRI using T_2_ contrast for delineation of the kidney from surrounding tissue. Top: T_2_ map and T_2_-weighted images of a mid-coronal oblique slice of a male rat kidney using a conventional multi spin-echo (MSME) technique (TR = 500 ms, number of echoes = 13, first TE = 6.4 ms, inter-echo time ΔTE = 6.4 ms, number of averages = 1, acquisition time = 58 s, in-plane spatial resolution = (226 × 445) µm^2^, FOV = (38.2 × 50.3) mm^2^, matrix size = 169 × 113 (zero-filled to 169 × 215), slice thickness = 1.4 mm, [37]) obtained at 9.4 T using a small bore animal MR scanner. Bottom: T_2_ map and T_2_-weighted images of a mid-coronal oblique image slice of male human kidney using a multi-echo, radially sample RARE variant (TR = 2000 ms, number of echoes = 12, inter-echo time ΔTE = 6.34 ms, number of averages = 1, number of excitations = 300, oversampling factor = 2, in-plane spatial resolution = (1 × 1) mm^2^, FOV = (256 × 256) mm^2^, matrix size = 256 × 256, slice thickness = 5.0 mm, number of excitations = 300, receiver bandwidth 810 = Hz/pixel, [129]) obtained at 3.0 T using a clinical MR system

Anatomic MRI of the kidney might appear at first glance to be an easy task. However, it is challenged by the competing constraints of scan time, spatial resolution, and image quality. The selection of an appropriate MRI protocol for kidney size quantification is not so much a matter of contrast mechanisms inherent to MRI and of MR pulse sequences. The boundaries of the kidney can be readily delineated for most endogenous MRI contrasts due to the high contrast-to-noise ratio between the kidney and surrounding tissues. This concerns contrast-weighted MRI or parametric mapping of specific metrics inherent to MRI. T_2_-weighting is a common approach for renal anatomic imaging used for segmentation of the kidneys (Fig. 1). T_2_-weighted MRI involving standard spin-echo imaging techniques, multi spin-echo techniques for parametric mapping of T_2_, and variants of Rapid Acquisition with Relaxation Enhancement (RARE) imaging provide excellent contrast-to-noise ratio between the kidney and surrounding tissues (Fig. 1). T_2_-weighting also provides sufficient intrarenal contrast to discriminate the renal layers. Adding fat saturation is beneficial to address chemical shift artifacts induced by adjacent peri-renal fat signals overlaying with the kidney. T_1_-weighted Dixon fat–water MRI is an alternative approach, which has been used for abdominal MRI in large-scale population studies [41, 42]. 

Arguably, the challenges of kidney size MRI arise more from the competing concerns of kidney coverage versus scan time, including the propensity to physiological motion. This relationship is more relaxed for single time point, snapshot acquisitions, which are common for current breath-held or free breathing renal MRI protocols. Kidney coverage versus scan time is a challenge for dynamic MRI monitoring of kidney size, which is an emerging application. Here, the temporal resolution of MRI constitutes a critical dimension for the assessment of abrupt changes in kidney size during acute scenarios.

### 3D MRI renal volume assessment

3D MRI affords full coverage of both kidneys with exquisite anatomical detail, in comparison to sonography, which is commonly used for measuring kidney size. Planning a 3D MRI slab that covers both kidneys is straightforward and does not require an expert reader. In current clinical practice, consistent automated 3D slab planning for abdominal and renal MRI is commonly achieved with state-of-the-art machine learning approaches that are included in the scanning protocols. This approach promotes reproducibility and consistent image quality, and ensures that measurements can be performed with minimal bias and low inter- and intra-operator variability. Recognizing this benefit, automated slab planning is employed in large-scale population imaging studies for kidney size assessment [41, 42]. 3D MRI with full kidney coverage is essential for the assessment of the total kidney volume (TKV). This creates substantial motivation for developing rapid 3D MRI techniques to overcome the image quality constraints of MRI of the abdomen attributed to respiratory motion artifacts. The most widely used approach to address respiratory motion-induced artifacts is to acquire 3D MRI data in breath-hold which is adopted in clinical studies. However, even under perfect breath-hold conditions, displacement of the diaphragm occurs throughout the breath-hold period which may result in motion-induced image artifacts. For free breathing MRI, respiratory motion is commonly dealt with using triggering, gating, pilot-tone navigators, registration-based motion correction, motion-weighted reconstruction, or motion-resolved reconstruction to compensate for respiratory motion-induced artifacts [43]. These approaches all require information about the respiratory motion pattern, are prone to irregular breathing and motion drifts, and are not contrast or signal intensity agnostic [44]. Recent reports have highlighted explorations into 4D MRI (three spatial dimensions + one time dimension) of the abdomen using under-sampled non-Cartesian MRI for acceleration [45]. An implementation of stack-of-stars golden angle radial sampling using under-sampled spokes in conjunction with GRASP Pro reconstruction enabled a temporal resolution as good as 0.63 s per 3D abdominal volume [46]. While these advancements are conceptually appealing for renal size assessment, they are thus far limited to proof-of-principle studies and have not yet been approved for clinical imaging.

### 2D MRI planimetry

The value of whole kidney coverage for kidney size assessment must be balanced with clinical feasibility. 3D MRI scans with whole kidney coverage are time-consuming, and prone to artifacts from respiratory motion. Long scan times pose a major impediment for dynamic and longitudinal clinical and experimental studies, and severely limit the potential for TKV assessment in translational research and routine clinical practice [47]. This constraint can be offset by 2D MRI in conjunction with planimetry of a central cross-section of the kidney, as opposed to whole kidney coverage MRI [6, 48–51]. Mid-slice planimetry is established in the literature for renal size assessment [28, 37]. A caveat of 2D MRI and planimetry is that it covers only a cross-sectional area rather than the entire renal volume. Therefore, renal size assessment from single slice 2D MRI may show lower sensitivity for changes in renal size, and may be less able to detect subtle alterations in renal size than renal volumetry. The volume can be approximated to scale with the third power of the radius (r) of the kidney, while the area deduced from cross-sectional 2D MRI is related to r^2^. It is reasonable to assume that subtle alterations in kidney size detected by 2D MRI planimetry would show more pronounced changes with 3D MRI volumetry, since the volume has a larger dynamic range for relative changes in kidney size. Deciding between 3D MRI volumetry and 2D MRI planimetry is not a fundamental issue for renal size assessment. Both can be converted if one assumes uniform shrinkage or swelling of the kidney along all three spatial dimensions, such that changes in kidney size along the third dimension are similar to the changes in the two spatial dimensions covered in 2D MR planimetry

1 $$\frac{\Delta TKV}{{TKV_0 }} = \left( {\frac{\Delta A}{{A_0 }} + 1} \right)^\frac{3}{2} - 1.$$

Whole kidney volumes can be also derived from 2D stereology, which involves 2D planimetry of a set of contiguous 2D slices followed by summing the products of the area measurements and the (nominal) slice thickness.

### 1D MR line-scanning for high-resolution monitoring of changes in kidney size

The sampling time and temporal resolution of conventional 2D MRI of the kidney may still be too coarse for detecting abrupt changes in kidney size, such as those seen in response to acute pathophysiological interventions. The temporal resolution of 2D MRI can be further reduced to the (sub)second range using rapid imaging or acceleration techniques, including parallel imaging and compressed sensing. Expanding from the acquisition of two-dimensional k-space data to the acquisition of a one-dimensional k-space line provides a viable alternative for further enhancing temporal resolution. This reduction in the number of dimensions is called line-scanning, and involves the acquisition of only one k-space line of interest. Line-scanning facilitates a temporal resolution as good as a few milliseconds, which is comparable with the sampling rate of readouts from invasive probes used in integrative physiology measurements. Line-scanning has proven its value for high-speed mapping of distinct functional response properties across human brain cortical layers [52]. The possibility of line-scanning across renal layers is conceptually appealing to pursue MR-based renal size assessment, but has not been explored so far. Due to the speed advantage, line-scanning is suited for (real-time) motion tracking [53, 54]. Hence, line-scanning could be used for simultaneous renal size assessment and for renal motion tracking. One concern with using MR line-scanning is that the reduction to one dimension sacrifices kidney coverage and resolution along the kidney boundaries, which may constrain the sensitivity to changes in renal size. However, closer examination reveals that this concern is unwarranted. Line-scanning MR of the kidney can be extended towards the acquisition of multiple lines with arbitrary orientation, to acquire profiles along the main axis of the kidney. This approach would approximate 2D planimetry using two lines aligned with the two main axes of the kidney. Even 3D volumetry can be approximated using three lines aligned with the main axes of the kidney. Line-scanning offers another advantage for renal size quantification, especially for acute interventions in renal physiology where size changes in the different layers are likely to be uniform. The spatial resolution of rapid 3D MRI or 2D MRI might be compromised due to scan time constraints. Signal decay due to relaxation processes may limit the spatial resolution for single shot techniques or for rapid imaging. With line-scanning, the spatial resolution can be enhanced by expanding the read-out window without significant costs in temporal resolution. The spatial resolution gains of line-scanning could be instrumental for improving the sensitivity and precision of renal size assessment, and demands further investigation.

To summarize, renal size assessment, especially for 1D and 2D MRI approaches, may be less challenging in kidneys with regular anatomy versus kidneys with disrupted anatomy such as in autosomal dominant polycystic kidney disease (ADPKD).

## What is the state-of-the-art of kidney segmentation?

Quantification of renal size requires accurate extraction of the kidney contour from the surrounding structures within the MRI data. The sum of all image voxels or pixels within the boundary of the kidney provides the kidney volume for 3D MRI, or the kidney area for 2D planimetry. Segmentation of the kidney from MRI may appear at first glance to be an easy task. It is, however, challenged by the need for speed, accuracy, automation, and reproducibility. Kidney segmentation presents several challenges related to intra- and inter-subject signal intensity differences and changes, intensity variations due to the diversity of the MRI techniques and contrast mechanisms used, and partial volume effects. The space and time dimensionality of a time series of MRI data constitutes another challenge for kidney segmentation, because signal intensity and image contrast may change dramatically during dynamic or longitudinal studies. Examples for this scenario include the severe reduction in signal and tissue contrast of T_2_-weighted MRI in the presence of (ultra)small iron oxide nanoparticle-based contrast agents used for rBVF assessment or during renal tissue hypoxia in comparison to healthy unmanipulated controls [55].

## Manual kidney segmentation

Manual tracing of the kidney contours for segmentation is time-consuming and highly prone to observer bias. The time needed for manual segmentation depends on the experience of the observers. Considering that the anatomy of the human kidney is more complex than that of the rodent kidney, a thorough manual segmentation of the human kidneys will likely require more time and observer experience than for rodents. Manual segmentation of human kidneys comparing beginner and expert readers was reported to require 26–35 min on average for 3D volumetry using common software tools such as ImageJ or Osirix, approximately 15 min for 2D stereology covering the entire kidney, ~ 9 min for 2D mid-slice planimetry, and approximately 5 min using the ellipsoid approach [6]. Another report documented the average time to measure TKV using manual segmentation and stereology as 44 ± 18 min [51]. Despite the considerable time demands of manual segmentation, it is still regarded in the community as the gold standard. Manual segmentation of kidney MRI data provides the foundation for meticulously annotated imaging data, and for establishing the ground truth for the training, evaluation, and validation of more sophisticated and modern renal segmentation approaches [56]. To establish the ground truth, a consensus reading that integrates the manual kidney segmentation of multiple independent observers provides an approach to improve the accuracy and precision of manual segmentation, and control interobserver discrepancies. A recent study conducted a consensus reading of manual segmentation of rat kidney from five independent observers for determining the ‘ground truth’ [37]. For this purpose, the median voxel count of the five observers was computed for each data set. Then, the renal segmentation of one observer was used as a starting point, with T_2_-maps presented to all observers simultaneously with a transparent overlay of the area determined as belonging to the kidney. Upon presentation of each T_2_ map and the corresponding transparent overlay, all observers agreed in real time to add or delete specific voxels from the overlay, to improve the accuracy of the renal boundaries. The result was a consensus on the total area assigned as renal tissue, which eliminated the bias of any individual observer. The changes were applied in real time, and a strict time limit of 90 s was established to achieve the consensus renal segmentation of each T_2_ map [37]. This strategy resulted in a negligible overall percentage difference versus the mean and median values of the five independent observers (mean 0.2 ± 1.0% and mean − 0.2 ± 0.9, respectively) [37]. These findings highlight the value of consensus reading of manually segmented kidney contours to determine an unbiased “ground truth” for comparison with automated approaches to kidney segmentation.

## Kidney segmentation using geometric models

The time constraints of manual segmentation can be circumvented using parametric modelling of the kidney with pre-established geometric shapes. The least complex approach for determining kidney volumes from multi-slice 2D or 3D MRI is to assess the main axes of the kidney to obtain the length, width, and depth of the kidney, and then apply the ellipsoid formula calculation: π/6 × length × width × depth. For this approximation, the length is determined from a sagittal view. The width and depth can be derived from the largest transverse diameter of the kidney. Kidney volume estimation using the ellipsoid formula is a common approach used clinically, due to its simplicity and speed, requiring less than 2 min for renal volume assessment. However, this approach over-simplifies the kidney structure, which is not a true ellipsoid structure, and thus, volumes calculated using the ellipsoid formula are not accurate. In vitro MRI evaluation revealed a 24% underestimation of the kidney volume derived from the ellipsoid formula [57]. The ellipsoid approach was reported not to be accurate enough to follow TKV changes in ADPKD patients over time, even masking the main clinical study finding [6].

**Fig. 2:**
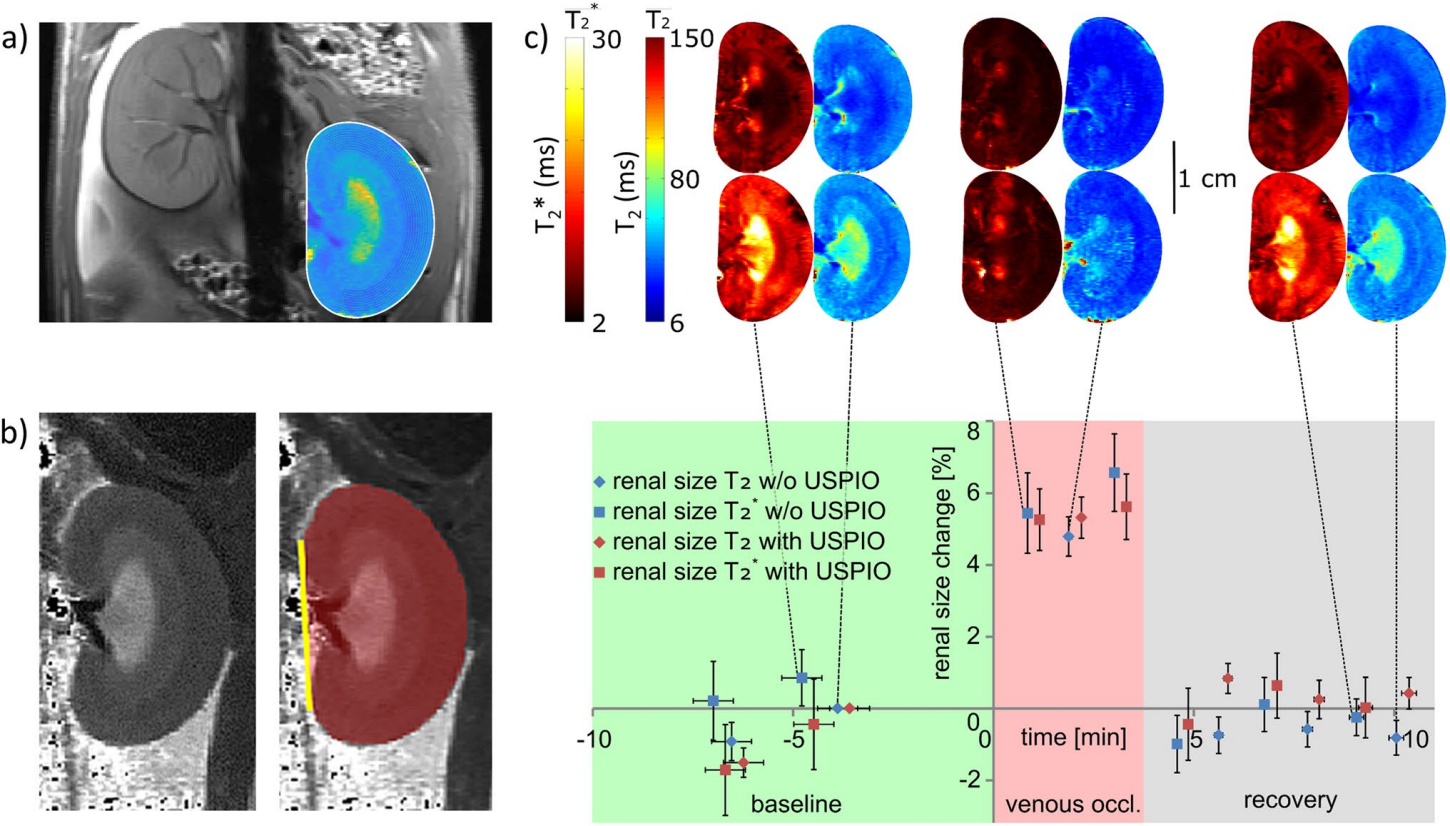
Illustration of the geometric automated bean-shaped model (ABSM) used for renal size quantification in rat kidney. **a** The kidney shape function obtained from the ABSM overlaid on the corresponding T_2_ map obtained for a rat kidney. The dashed white line indicates the kidney surface. **b** Exemplary gray scale coded T_2_ map of a rat kidney and the corresponding kidney size quantification obtained from 2D planimetry of a mid-coronal slice. **c)** Time course of relative changes in renal size (mean ± SEM, *n* = 12) during the occlusion of the left renal vein, and upon cessation of the occlusion, derived from T_2_ and T_2_* mapping of the rat kidney using the ABSM. The intervention was performed twice, i.e., before (blue symbols) and after (red symbols) administration of the ultrasmall superparamagnetic iron oxide (USPIO) preparation, ferumoxytol. Exemplary color-coded renal T_2_*- and T_2_-maps (color scales in milliseconds) are shown in the upper panel, prior to (lower row) and following (upper row) administration of the USPIO [37]

Hybrid level-set methods provide an extension of renal segmentation using geometric shapes [47–50]. These approaches are semi-automated, and they usually require an expert observer to manually pre-select the measurements, thresholds, and definition of landmarks or seeds, to initialize the models. Recently, a preclinical study demonstrated the feasibility of automated segmentation of the mid-slice cross-sectional area of the rat kidney using a geometry-based bean-shaped model (ABSM) [37]. An analytic function describing the shape of the kidney was used, and fitted to the edges found in the 2D MR images (Fig. 2) [37]. This approach required less than 14 s processing time per mid-slice cross-sectional T_2_ or T_2_* map. This gain corresponds to a ~ 70-fold increase in segmentation speed using a standard consumer PC, compared to the time required by trained human readers. Parallel computing, GPU implementation, or high-performance computing hardware would further accelerate ABSM-based quantification of kidney size. Crucially, the ABSM model yielded a high level of both accuracy and precision, equivalent to the ground truth derived from manual segmentation. The effectiveness of the ABSM for high time-resolved quantification of renal size in rats was demonstrated in the context of (patho)physiologically relevant interventions mimicking clinical scenarios, as illustrated for venous occlusion in Fig. 2 [37]. Additionally, the contrast and signal agnostic efficacy of the ABSM was demonstrated using administration of the USPIO preparation ferumoxytol, which covers a large dynamic range of MRI signal-to-noise ratios and contrast-to-noise ratios, exceeding that induced by pathophysiologically relevant interventions or using other MR contrasts aside from T_2_ (Fig. 2). Monitoring kidney size derived from the ABSM allowed accurate physiological interpretation of renal oxygenation changes in acute pathophysiological scenarios [37, 58].

## Machine learning-assisted kidney segmentation

**Fig. 3:**
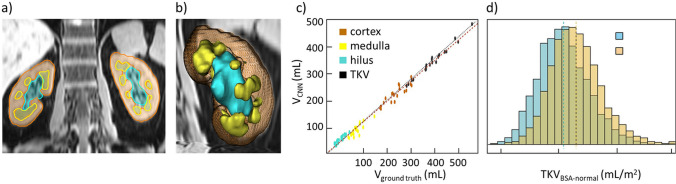
Automated segmentation of the human kidney and its compartments—cortex, medulla, hilus, and cysts—using a convolutional neuronal network on 3D MRI data. **a** T_1_-weighted Dixon water-only image (spatial resolution 1.4 × 1.4 × 3 mm^3^) in a 2D coronal view of the kidney. Cortex, medulla, and hilus are shown as colored overlays in red, yellow, and turquoise. **b** 3D rendering of the left kidney shown in **a**. **c** Comparison of the total kidney (TKV), cortex, medulla, and hilus volumes predicted by the CNN (y-axis) versus the ground truth volumes obtained from manual segmentation (x-axis). Dashed lines were obtained from linear model fits. **d** Distribution of total kidney volume normalized to body surface area (BSA) (blue = female, orange = male). After correction for BSA, males have higher total kidney volumes compared to females. Adapted from [109]. Courtesy of Peggy Sekula, Institute of Genetic Epidemiology, Faculty of Medicine and Medical Center—University of Freiburg, Freiburg, Germany

Machine learning (ML) provides a viable alternative for manual segmentation, and has gained momentum for automated kidney size quantification. Machine learning-based renal segmentation provides a solution for deforming the kidney shape using a constrained statistical model-based algorithm trained upon a dataset [59]. This approach requires minimal user interaction. Out of the various fully automated supervised and unsupervised ML approaches that have been explored, deep-learning (DL) algorithms, particularly Convolutional Neural Networks (CNN), have become prevalent for kidney segmentation [59–64]. Recent advances in CNN-based renal segmentation reported processing times as good as 1 to 10 s per subject—excluding the time and effort needed to obtain meticulously annotated training data, setup, and train the model [64–66]. An example of fully automated human kidney segmentation from T_1_-weighted Dixon water and fat images using a hierarchical, multiscale 3D CNN framework is presented in Fig. 3. The prediction of a full volume required less than 2 min per subject, using a 16-core machine without GPU support. The CNN was validated against the ground truth obtained from manual segmentation and provided a dice coefficient of 0.95 (Fig. 3). A neural network based on a 2.5D U-Net variation was used for kidney volume measurements in 40,000 subjects of the UK Biobank study [65]. Validation of the neural network showed a Dice coefficient of 0.95 which was similar to the Dice coefficient of 0.96 obtained for repeated segmentation by one human operator [65]. Owing to the speed of the neural network, this large-scale data set can be processed within 1 day, yielding volume measurements for left and right kidney [65]. Clinical implementation of an AI algorithm tailored for MRI-based TKV quantification was recently demonstrated for ADPKD [67]. In this interdisciplinary study, AI-based TKV quantification showed high levels of agreement with manually edited kidney segmentation and was non inferior to interobserver variability [67]. The assessment of dynamic changes in TKV requires low measurement variability and high precision. To meet this requirement a 3D multi-modality, multi-class segmentation model was implemented using multiple endogenous MRI contrasts (T_1_, T_2_, SSFP, and DWI) and CT data [68]. With this multi-modality approach a 1.3% test–re-test reproducibility of TKV was achieved [68].

The development, training, and validation of DL algorithms tailored for automated kidney segmentation may suffer from small data sample sizes and from data biases (for example, male versus female subjects). To address these constraints, generative adversarial networks (GANs) can be used to generate synthetic images. Recently, GANs were used to generate synthetic images from T_2_-weighted MRI of the kidney simulating the precontrast, corticomedullary, early nephrographic, and nephrographic phases of multi-phase, contrast-enhanced MRI examinations [69]. This style transfer approach from T_2_-weighted MRI of the kidney achieved high performance for renal segmentation in different dynamic phases of the original multi-phase contrast-enhanced MRI acquisitions [69].

**Fig. 4:**
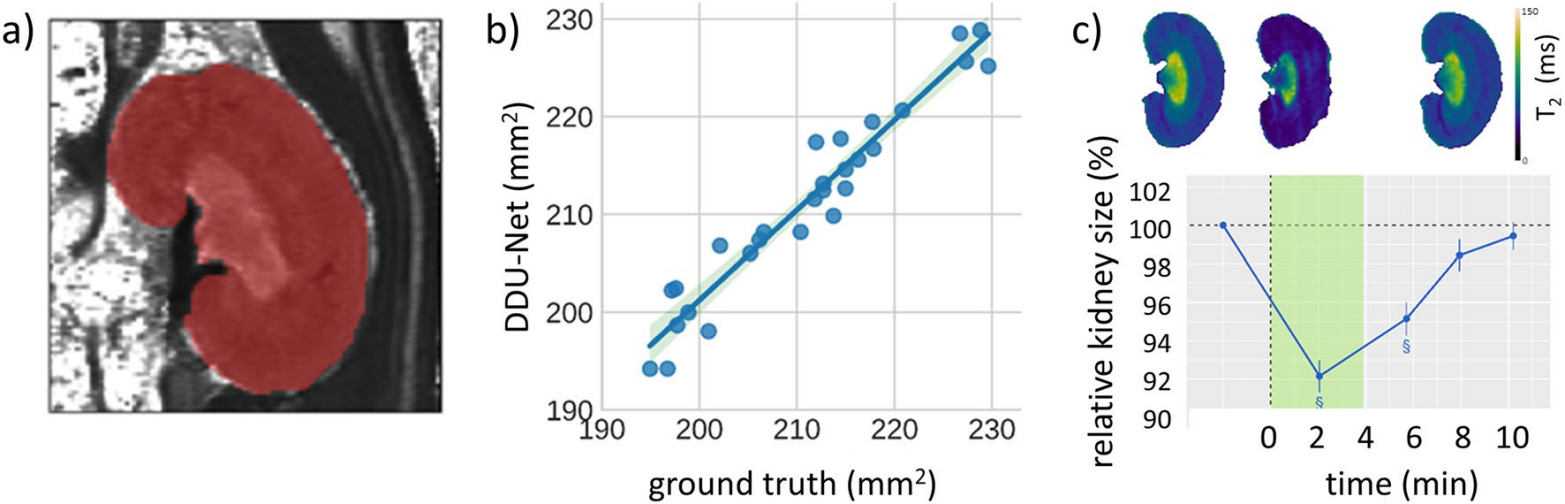
Automated rat kidney segmentation and size quantification using a deep dilated neuronal network (DDU-Net) for segmentation of 2D data sets obtained from T_2_-maps [70]. **a** Exemplary T_2_ map and the corresponding kidney size prediction obtained from the DDU-Net. **b** Comparison of the DDU-Net with the ground truth obtained from manual segmentation using the consensus of five readers. Linear regression plots (± 95% confidence interval) are shown for the three baseline timepoints. **c** Relative kidney size over time obtained upon occlusion of the suprarenal aorta. Top: Exemplary T_2_-maps are shown before (left) and during (center) the intervention, and after recovery (right). Bottom: Time course of kidney size changes (mean ± SEM, *n* = 12), relative to the baseline (211 ± 4 mm^2^). The duration of the occlusion is highlighted in green. Reproduced and adapted from [70]

A recent preclinical study employed dynamic parametric T_2_ mapping of the kidney in rats in conjunction with a custom-tailored deep dilated U-Net (DDU-Net) architecture, which was trained and validated against ground truth from manual segmentation and benchmarked against an analytical segmentation model (Fig. 4) [70]. For the DDU-Net, a Dice coefficient of 0.98 compared to the ground truth obtained from manual segmentation and a CPU computation time of ~ 70 ms per segmentation were reported. The GPU implementation of the DDU-Net reduced the computation time per segmentation to 10 ms. The DDU-Net was applied in an in vivo longitudinal MRI study in rats to monitor changes in renal size upon interventions that emulate clinically relevant scenarios [70]. During occlusion of the suprarenal aorta, the DDU-Net detected a reduction in kidney size of -8 ± 1%, while renal venous occlusion resulted in an increase of 5 ± 1%. Additionally, the DDU-Net detected a modest increase of 2 ± 1% upon administration of furosemide, whereas hypoxemia induced a slight decrease of -2 ± 1%. X-ray contrast agent-induced acute kidney injury exhibited the most significant change in kidney size, an increase of 11 ± 1%, as assessed by the DDU-Net (Fig. 4).

In general, deep-learning-based kidney segmentation from MRI requires an adapted design and training regimen, because each dataset presents unique challenges in terms of image quality, resolution, contrast, and anatomic variations. These variations necessitate customized preprocessing steps, network architectures, and training strategies to ensure accurate segmentation performance across diverse datasets. Additionally, factors, such as MRI pulse sequences, reconstruction techniques, and hardware conditions, further emphasize the need for dataset-specific optimization to achieve reliable and robust segmentation results. These challenges can be addressed with self-adaptable U-Nets, a deep-learning-based segmentation approach that automatically configures itself for any new task, including preprocessing, network architecture, training, and post-processing [71]. The self-configuring no new U-Net (nnU-Net) framework has seen a wide range of segmentation applications including snapshot images and imaging time series of the kidney [70–72], rendering DL-based segmentation of the kidney instrumental for a broad end-user group with standard computing resources, and without the need for expert knowledge. Owing to this simplicity, nnU-Net was deployed for deep-learning-based abdominal organ segmentation on 20,000 whole-body MRI datasets from the UK Biobank (UKBB) or the German National Cohort (NAKO) epidemiological studies, including large-scale visual expert quality assessment of these segmentations, analysis of factors that impact segmentation quality ratings, and characterization of image-derived phenotypes based on segmentation quality [73]. The results obtained from this evaluation showed the feasibility of large-scale, DL-based kidney segmentation of MRI data with high overall accuracy. Notwithstanding this progress, this study highlighted the critical role of visual quality control of segmentations derived from nnU-Net to ensure the validity of down-stream analyses in large epidemiological imaging studies [73]. Recent work on monitoring renal size in a longitudinal MRI study in small rodents concluded that a rather limited and challenging dataset requires a customized solution beyond the scope of self-adapting approaches, especially when in vivo experiments lead to a data shift [70]. Thus, several characteristics could be added to the portfolio of nnU-Net self-configuration including increased depth of the network, additional dilated convolutions, intra-level skip connections, concatenation of the layer output, and downsampling via strided convolution. It is expected that incorporating these configurations into the framework may allow the nnU-Net to address a broader range of challenges and improve performance, as has already been partially implemented [70, 74].

## What are the opportunities of preclinical kidney size monitoring?

The fact that changes in kidney size indicate pathophysiologic developments has been demonstrated by MRI in a variety of experimental models. Preclinical studies emulating clinical conditions such as acute obstructions of the urinary tract due to urolithiasis, or during upper urinary tract endourologic procedures, have demonstrated that the ensuing congestion results in a TVF increase [75, 76]. Studies emulating administration of X-ray contrast media for cardiac procedures have demonstrated an increase in TVF induced by the high viscosity [76, 77]. Administration of furosemide, a loop diuretic that increases TVF by lowering tubular water resorption also resulted in a subtle increase in kidney size in rats [76]. Studies emulating clinical procedures such as clamping of the suprarenal aorta or renal artery during surgery revealed decreases in kidney size related to a decrease in BVF, while clamping of the renal vein showed increased kidney size related to an increase in BVF [37, 76–78]. Acute hypoxemia, which occurs in clinical scenarios with decreased hematocrit or reduced pulmonary O_2_ diffusion, was also shown to decrease kidney size in rats, probably due to reduced BVF [37, 76–78]. Recent studies have incorporated MR-based kidney size assessments in several animal models of renal pathophysiology, including diabetes, PKD, chronic ureteral obstruction, and renal allograft transplantation [62, 79–84].

Our preclinical studies recently demonstrated that MR-based assessment of changes in kidney size is crucial for correct physiological interpretation of MRI-based assessments of renal tissue oxygenation obtained by blood oxygenation level-dependent (BOLD) MRI techniques. Renal tissue hypoxia is a pivotal early element in the pathophysiology of acute kidney injury (AKI) and its subsequent progression to chronic kidney disease (CKD). Hypoxia also plays a major role in the pathophysiology of diabetic kidney disease (DKD) [30, 85–92]. Therefore, assessment of renal oxygenation by BOLD-MRI could become a vital assay for research into renal (patho-)physiology and for clinical application. This approach relies on the fact that deoxygenated hemoglobin (deoxyHb) is paramagnetic, and therefore impacts the MRI relaxation times T_2_* and T_2_. Both T_2_* and T_2_ decrease with increasing deoxyHb concentration. Thus, T_2_* and T_2_ can provide a surrogate marker of tissue oxygenation, due to their dependence on the O_2_ saturation of hemoglobin (StO_2_) and its relationship to the partial pressure of O_2_ (pO_2_) in blood and tissue [2, 93]. However, T_2_* and T_2_ reflect the amount of deoxyHb per tissue volume; therefore, the relationship between renal T_2_*,T_2_ and tissue pO_2_ is also dependent on the renal BVF and TVF [2, 31, 78, 93, 94].

**Fig. 5:**
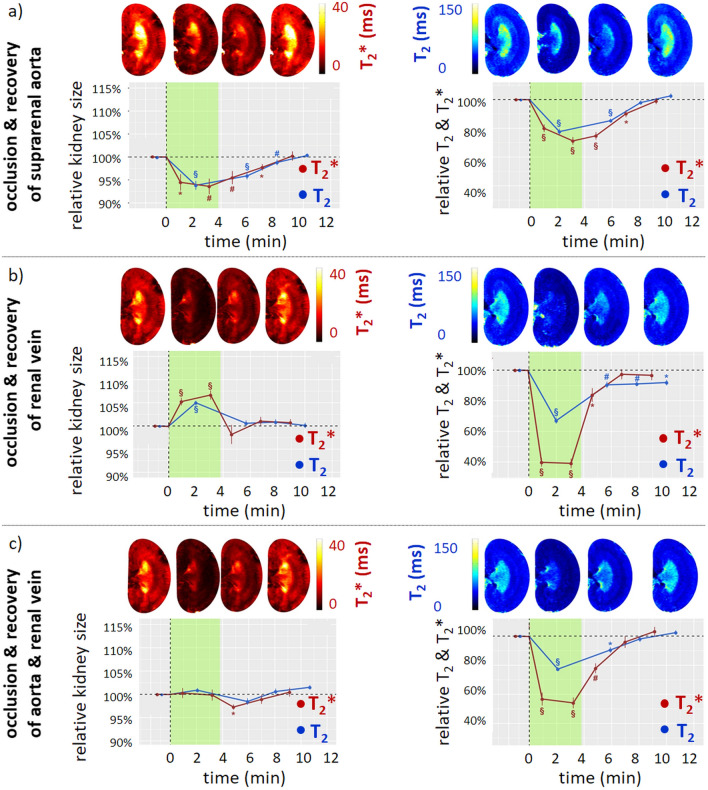
Temporally resolved changes of kidney size during acute interventions. **a** Time courses during occlusion of the suprarenal aorta and recovery**.** Exemplary T_2_* (left) and T_2_ (right) maps obtained for a rat kidney in vivo. Time course of relative changes (mean ± SEM) for kidney size (cross-sectional area) and T_2_ (blue) and T_2_* (red) obtained for cortex before the intervention (baseline), during the intervention (green area), and during recovery. **b** Time courses during occlusion of the renal vein and recovery. Exemplary T_2_,T_2_* maps obtained for a rat kidney in vivo. Time course of relative changes for kidney size and T_2_,T_2_* derived from the cortex. Colors, absolute baseline values, and significance signs as in **a**. **c** Time courses during simultaneous occlusion of the aorta and the vein and recovery. Exemplary T_2_,T_2_* maps obtained for rat a kidney in vivo. Time course of relative changes for kidney size and T_2_,T_2_* derived from the cortex. Colors, absolute baseline values, and significance signs as in **a**. For all interventions, the automated bean-shaped model was used for kidney segmentation and quantification of changes in kidney size. *P < 0.05; ^†^*P* < 0.01; ^‡^*P* < 0.001. Reproduced and adapted from [58]

Recognizing that events leading to acute renal hypoxia are often associated with changes in BVF or TVF, and that these changes are accompanied by changes in kidney size, we used dynamic MRI to monitor kidney size in parallel with T_2_*,T_2_ mapping in rats (Fig. 5). This was done during clinically realistic interventions that alter renal tissue oxygenation in a reversible manner. These interventions include brief occlusion of the suprarenal aorta (OA), the renal vein (OV), or both (OAV), in addition to interventions with longer lasting effects, including injection of an X-ray contrast medium [76]. As shown by Fig. 5, OA resulted in a decrease in kidney size and a moderate decrease in T_2_*,T_2_. OV resulted in an increase in kidney size and a much more pronounced decrease in T_2_*,T_2_, while OAV left kidney size unchanged and resulted in an intermediate decrease in T_2_*,T_2_ (Fig. 5) [76]. Previous studies with ‘gold standard’ invasive probes showed an equivalent decrease in tissue pO_2_ upon each of these three occlusions [95]. The reason for this discrepancy is that the changes in T_2_*,T_2_ reflect changes in the amount of deoxyHb per tissue volume, rather than directly measuring StO_2_. In addition to decreased StO_2_, intrarenal blood volume is reduced upon OA, increased upon OV, and unchanged upon OAV. Thus, the correct interpretation of T_2_*,T_2_ as surrogate markers for acute changes in renal tissue oxygenation must also take into account changes in kidney size. If T_2_*,T_2_ decrease and kidney size remains unchanged, tissue oxygenation is reduced. If T_2_*,T_2_ decrease and kidney size also decreases, the pO_2_ reduction is more severe than if kidney size is unchanged; if T_2_*,T_2_ decrease and kidney size increases, the pO_2_ reduction is less severe. We introduced a biophysical model to estimate changes in StO_2_ from changes in T_2_* and kidney size that yielded physiologically plausible calibration ratios for T_2_* [76].

Administration of an X-ray contrast medium induced a sustained kidney increase, with an initial increase in T_2_*,T_2_ followed by a moderate decrease [76]. Measurements by ‘gold standard’ probes showed an immediate, massive, and sustained drop in pO_2_ [96]. The explanation for this apparent discrepancy becomes clear from the observed increase in kidney size, which reflects the ‘compartment syndrome’ that results in compression of the intrarenal vessels and thus in decreased deoxyHb. This result underscores how MRI-based measurement of renal oxygenation by T_2_*,T_2_ is crucially dependent on monitoring accompanying changes in kidney size. Taken together, these preclinical findings demonstrated that monitoring kidney size allows an appropriate physiological interpretation of acute renal oxygenation changes obtained by T_2_*,T_2_ mapping [76].

Measurements of acute changes in kidney size alone cannot differentiate between changes in the renal BVF from changes in the TVF. However, this distinction will often be clear from the specific intervention performed in preclinical experiments, and will also be obvious in many clinical scenarios with acute kidney size changes. Furthermore, advanced MR methods support monitoring of acute changes in the TVF using diffusion-weighted imaging including pharmacological renal perfusion modulation, and in the BVF by use of contrast media such as ultrasmall superparamagnetic iron oxide [31, 55, 75, 97–100]. To summarize, the accelerating pace of discovery is driving the transfer of the insights deduced from renal size monitoring in experimental models into the clinical arena.

## What are the clinical opportunities of kidney size quantification?

An increasing body of literature outlines the potential of non-invasive imaging for evaluating renal size as a clinical parameter in the diagnosis, treatment monitoring, and prognosis in renal disease. In patients with polycystic kidney disease (PKD), kidney size correlates with disease progression and a decline in the glomerular filtration rate [28, 101, 102]. Consequently, the U.S. Food and Drug Administration and the European Medicines Agency include kidney size as a prognostic biomarker for use in clinical trials of new therapies for ADPKD [103, 104]. Detecting kidney size reduction due to parenchymal atrophy, sclerosis, and fibrosis has long been recognized as a tool to identify chronic kidney disease (CKD) and to determine its severity [105, 106]. Kidney size measured from ultrasound images is currently included as a prognostic imaging biomarker for diabetic kidney disease [107]. A recent position paper from the European Cooperation in Science and Technology Action PARENCHIMA included longitudinal monitoring of kidney size from MR images as a key measure for several renal diseases including ADPKD, hyperfiltration in early diabetic nephropathy, renal transplants, renal artery stenosis, and vesicoureteral reflux [108].

Large epidemiological studies such as the UKBB and NAKO provide an unprecedented repository of MRI and other health-related data of the general population, with the aim to better understand determinants of health and disease [41, 42, 65]. Population-scale, MRI-based automated kidney segmentation revealed an increased TKV, renal cortex, and hilus volume for male versus female subjects, while this difference was not observed for the medulla (Fig. 4) [41, 109]. The same study showed differences in body surface area (BSA) normalized volumes of the left versus the right kidneys for TKV and all renal layers (Fig. 4) [65, 109]. Age was inversely correlated with BSA-normalized TKV, with cortex and especially with medulla volumes, but was positively correlated with hilus volumes [109]. Conversely, estimated GFR was positively correlated with TKV, cortex, and medulla volumes, but inversely correlated with hilus volume. These epidemiological studies offer a springboard for further research into the stages and evolution of renal diseases over time, and responses to therapy and intervention [109].

Considering that sonography is readily available in most clinical settings and is inexpensive compared to MRI, utilizing MRI instead of ultrasound for the sole purpose of assessing kidney size in patients will most likely remain an exception. The major advantage of MRI is that it enables the assessment of several macroscopic and mesoscopic characteristics—and most importantly also functional characteristics—of the kidney, in a single MR examination.

Given the pivotal role of renal tissue hypoxia in the pathophysiology of AKI and its progression to CKD, as well as in the pathophysiology of CKD of other origins, BOLD-MRI is a prime candidate for parallel assessment of kidney size. The diagnostic potential of BOLD-MRI has been assessed in several patient cohorts, including patients with CKD, and in patients following kidney transplantation, among others [110–117]. In general, these studies have produced rather inconsistent results. Apart from using different MRI acquisition and analysis protocols, the main reasons for these inconsistencies include disease-related reductions in the BVF, including long-term effects due to rarefication of vessels in fibrosis or reduced vascular lumen due to vascular remodeling, as well as acute reductions due to interstitial edema formation or tubular obstructions that lead to intrarenal compartment syndrome [2, 31, 94]. As demonstrated by our preclinical studies, simultaneous assessment of changes in kidney size that accompany changes in either the BVF or TVF will potentially allow for more accurate (patho-)physiological interpretation of BOLD-derived measurements of renal oxygenation [76]. Phase-contrast MRI allows the assessment of renal blood volume in individual kidneys and has potential to provide complementary information for (patho-)physiological interpretation of changes in kidney size [118, 119].

If the reasons underlying a change in kidney size (i.e., changes in TVF or BVF) are not obvious from the respective clinical scenario alone, this can be determined by applying advanced MRI techniques that can be readily included in the patient examination, such as diffusion-weighted imaging or contrast-enhanced MR [31, 55, 75, 120–122]. Advanced MR methods can also be used to identify the degree of fibrosis, which is particularly important in severe CKD and is therefore a highly desirable clinical diagnostic parameter, even outside the context of BOLD [123, 124].

## Where is MRI of kidney size heading to?

The advances in renal size quantification have already generated intense interest for (pre)clinical application, and this is just the start. So far, MRI-assisted renal size quantification has mainly involved retrospective monitoring of changes in kidney size, where the MRI data are used off-line as an input for custom-made analysis software, and where the subject under investigation has already left the scanner—if not the facility or the hospital. Imagine if physicians and other end users could get MRI-assisted measures of renal size with relevant diagnostic signatures on-the-fly, before the MRI session is finished. Future developments should push towards prospective and real-time application of renal size quantification. For this purpose, deep-learning approaches tailored for kidney size quantification should be incorporated into the image reconstruction and post-processing pipeline of the MR scanner. Here, GPU implementations offer a way to improve computation time substantially compared to the conventional CPU implementations. These computation time improvements align with the temporal resolution of MRI and render on-the-fly assessment of kidney size feasible. Supported by harmonization of MRI acquisition protocols, these advancements provide unique opportunities for the MR vendors to integrate renal size application packages in their products, similar to current DWI, DCE, ASL, or fMRI application packages.

**Fig. 6:**
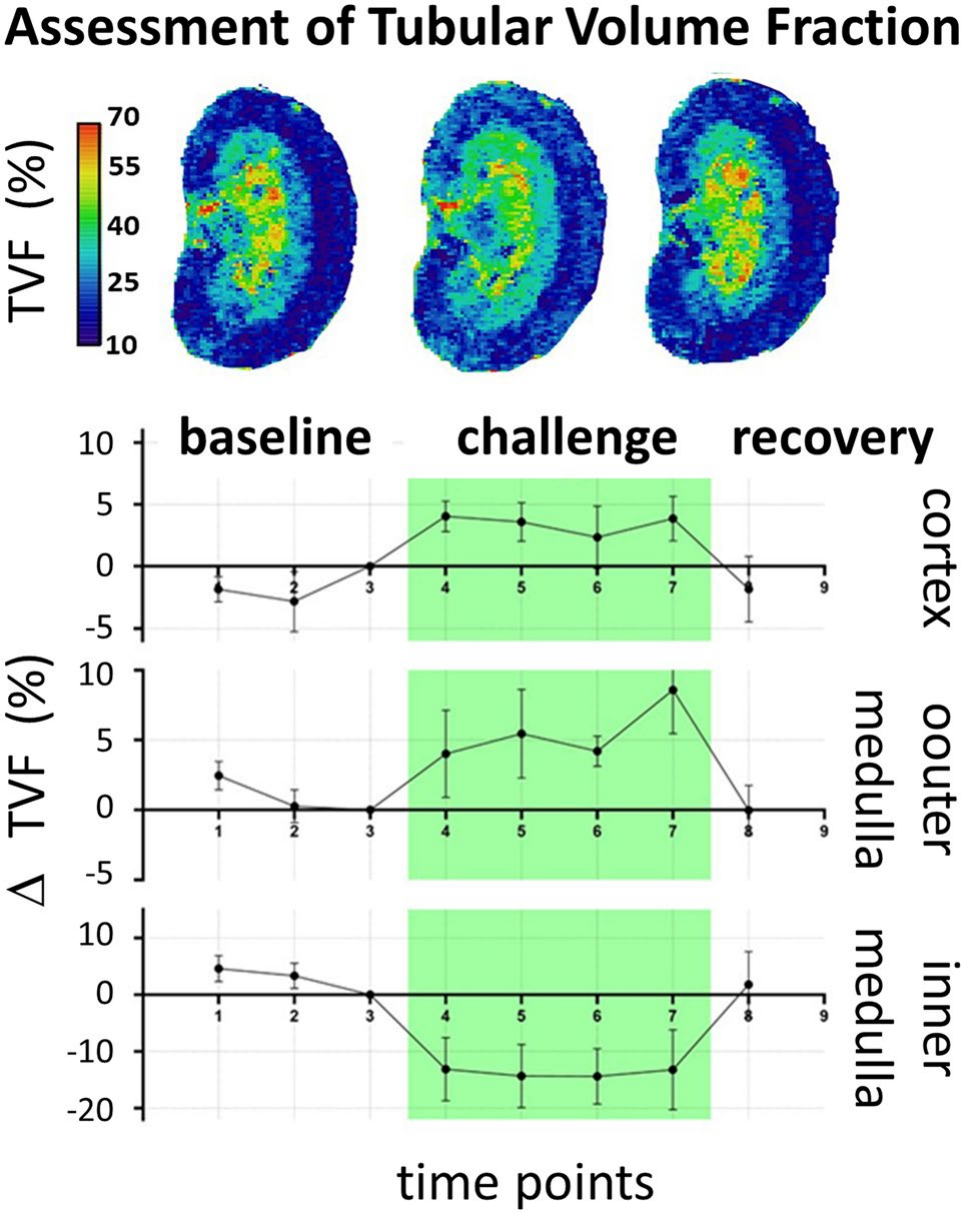
Time course of the tubular volume fraction (TVF) upon increased renal pelvis/tubular pressure [75]. Top: Exemplary TVF maps obtained for a rat kidney in vivo using decomposition of the bi-exponential T_2_-decay. Bottom: relative TVF changes (mean ± SEM) for cortex, outer medulla, and inner medulla, before the intervention (baseline), during the intervention (green area), and during recovery. Reproduced and adapted from [75]

The overall progress and the growing spectrum of renal size monitoring are encouraging. Yet, the ultimate potential is far greater still. While alterations in the tubular, vascular, and interstitial volume fractions often manifest as changes in kidney size, acute size measurements alone cannot distinguish between changes induced by altered blood volume *versus* altered tubular volume. This distinction requires further developments into mapping the renal BVF and the TVF. A recent study demonstrated the feasibility of intravascular contrast-enhanced MRI for monitoring rBVF, using these measurements to correct renal T_2_* for BVF variations [55]. This approach is now used in clinical studies in human CKD [120]. Tasbihi et al. entered an entirely new field of research on kidney health and disease by showing proof-of-principle dynamic T_2_ mapping of the MRI relaxation time for TVF cartography, and for monitoring TVF changes in rats (Fig. 6) [75]. For this purpose, the amplitude of the long T_2_ component was used as a surrogate for TVF, by applying multi-exponential analysis of the T_2_-driven signal decay. For the first time, this approach facilitated parametric maps of the TVF to be obtained in vivo under normal baseline conditions, and upon clinically realistic increases in renal pelvis and tubule pressure (Fig. 6) [75]. These are important steps towards deciphering the underlying (patho)physiological mechanisms behind changes in kidney size.

MRI of kidney size matters. In the majority of the literature, kidney size measurements are based on the entire renal capsule. Why is there a need for kidney sub-segmentation? Alterations in the tubular, vascular, and interstitial volume fractions leading to changes in the kidney size cannot be assumed to be uniform across the whole kidney. Further subdivision into the cortex, outer medulla, and inner medulla may be advantageous, and could lead to a more meaningful analysis of the factors that cause changes in kidney size. One example is the changes in TVF induced by intervention that increases tubular pressure [75]. This study showed a significant decrease in the TVF within the inner medulla during the pressure intervention, which returned to baseline levels during the recovery phase as outlined in Fig. 6 [75]. Looking closely at the dynamic T_2_ mapping data obtained during baseline, pressure increase, and recovery, the investigators noted an expansion of the renal pelvis during the intervention (Fig. 6). The increase in intrapelvic pressure during the procedure was strong enough to stretch the rather rigid tissue wall of the pelvis and compressed the comparatively softer tissue of the inner medulla, which is enveloped by the pelvis. This highlights the benefits of subdividing the renal compartments, since this result may not have been detected if only the size of the entire kidney was taken into consideration. Sub-segmentation can provide deeper insights into the biomechanical interactions between the different renal layers, as each kidney layer has its own specific elasticity and structure. Therefore, assessment of the kidney size with sub-segmentation can lead to more meaningful physiological interpretations.

**Fig. 7:**
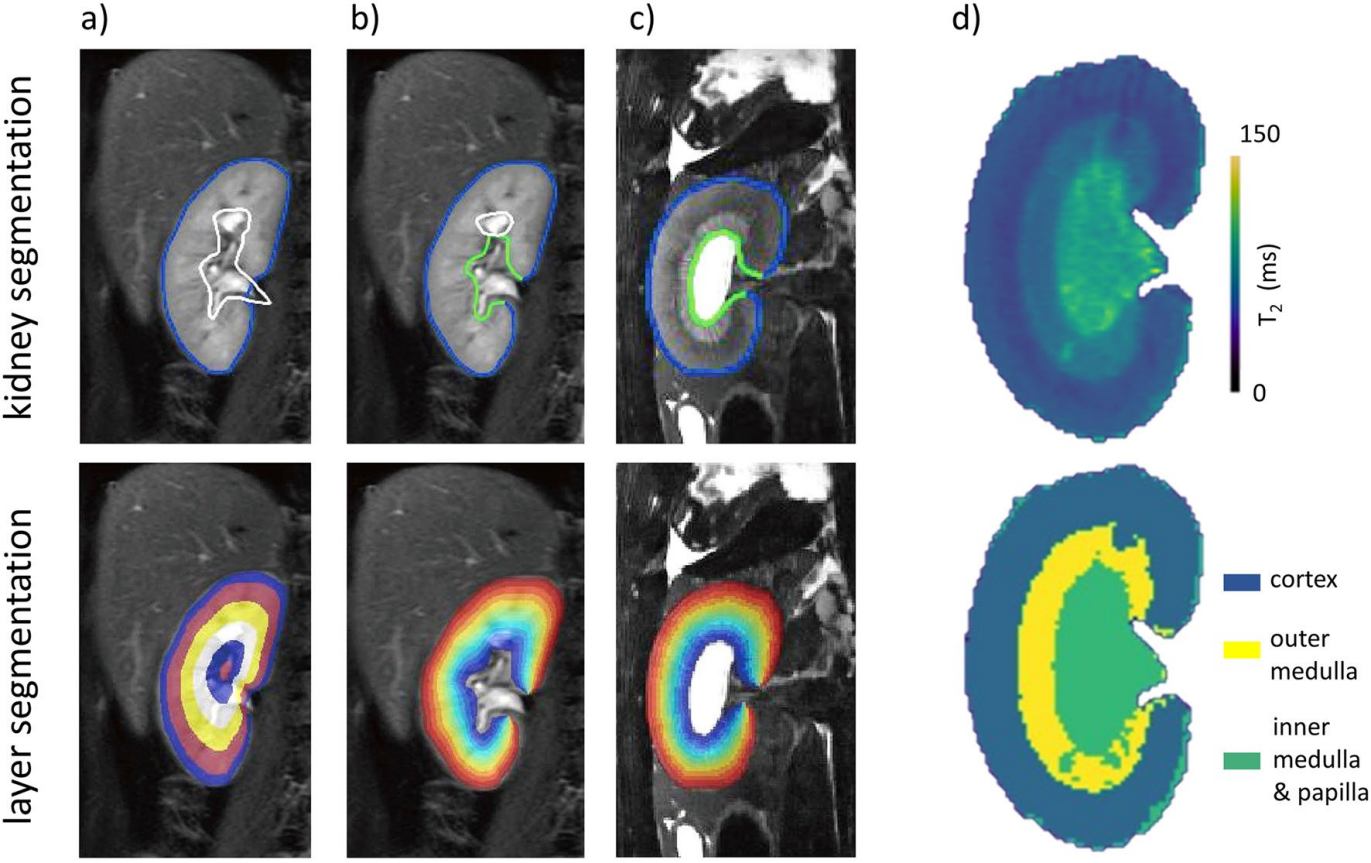
Global segmentation and layer-specific segmentation of the human and rat kidney. **a** Global and layer-specific segmentation of a human kidney using the concentric objects method. This approach uses the distance from the surface to the kidney ‘center’ to divide the renal parenchyma into a series of layers. **b** Semi-automatic global and layer-specific segmentation of a human kidney using the 12-layer concentric objects (TLCO) technique. The TLCO approach divides the renal parenchyma into 12 layers with equal-thickness, using the outer and inner boundaries of the kidney. **c** Semi-automatic global and layer-specific segmentation of a rat kidney using the TLCO approach. **d** Top: global segmentation of a rat kidney predicted by the DDU-Net using T_2_-maps. Bottom: layer-specific segmentation of a rat kidney derived from unsupervised machine learning using a Bayesian Gaussian mixture model

**Fig. 8:**
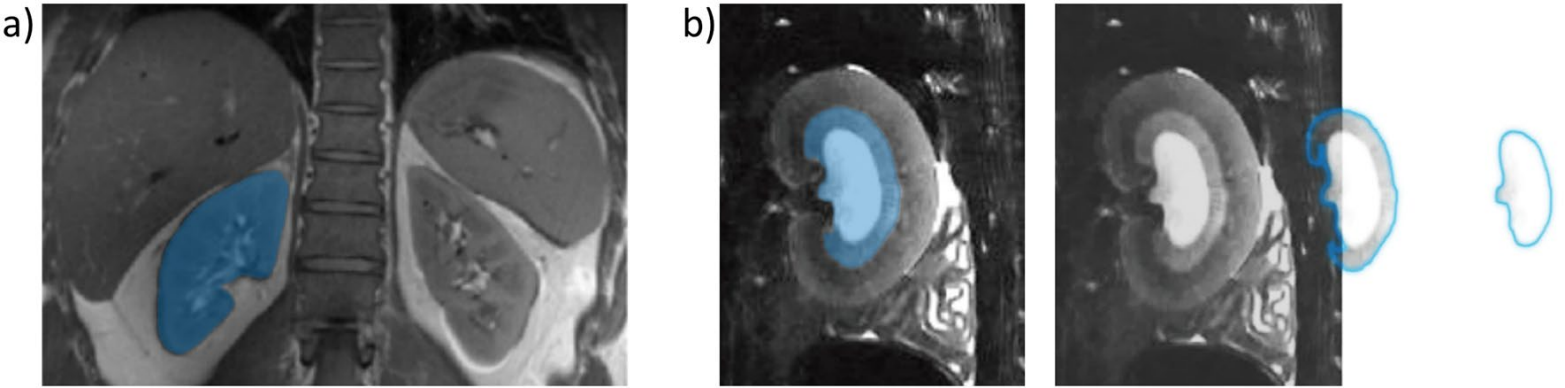
Kidney segmentation derived from the foundation model “Segment Anything Model” (SAM, https://segment-anything.com/). **a** Segmentation of the human kidney shown in the bottom of Fig. 1. The model has problems to correctly remove large vessels from the human kidney. **b** Layer-specific segmentation of the rat kidney shown in Fig. 7d. These masks can be used as inputs for further analysis of size changes in kidney compartments

Reconciling global renal size changes with alterations in the size of specific renal layers warrants further segmentation of the renal layers, along with segmentation of the renal contours. The 12-layer concentric objects (TLCO, [112, 115]) technique is a semi-automatic renal segmentation method, which is an advanced variant of the concentric objects (CO) method introduced by Piskunowicz et al. [125]. While the CO method takes the distance from the surface to the kidney ‘center’ into account to divide the renal tissue into a series of onion peels-like layers, the TLCO approach divides the renal tissue into 12 layers with equal-thickness, using the outer and inner boundaries of the kidney. The TLCO approach improves the precision of layer segmentation over the CO approach, especially in the human kidney with its complex vascular structure. Both the CO and TLCO approaches have been shown to enhance reproducibility of kidney segmentation for evaluating quantitative MRI biomarkers [126]. Examples obtained for human and rat kidneys using the CO and TLCO approach are shown in Fig. 7. A deep-learning-based framework for fully automatic TLCO has been established by Ishikawa et al. via a CNN and a conventional computer vision method to automatically delineate the outer and inner boundaries of the kidney [127]. Unsupervised DL algorithms are also conceptually appealing for assessing changes in the size of specific renal layers. Preliminary results obtained for layer-specific segmentation with unsupervised learning using a Bayesian Gaussian mixture model are shown in Fig. 7. The next steps into the future of segmentation of the kidney and its layers are already beckoning on the horizon in the form of foundation models trained on broad data to be applied across a wide range of use cases. One intriguing example is the “Segment Anything Model” (SAM, https://segment-anything.com/). SAM enables zero-shot generalization to unfamiliar objects and images, without requiring additional training. Driven by curiosity the authors used SAM to setup quick segmentation masks of the kidney (Fig. 8). A closer examination revealed that SAM still has problems to correctly remove large vessels from the mask of the human kidney, which constitutes a confounder for TKV assessment. Interestingly SAM provides reasonable results for layer-specific segmentation masks for the rat kidney which can be used as inputs for further analysis of size changes in kidney layers (Fig. 8).

The progress towards MRI of kidney size has been exciting, and the increasing recognition of its potential has been thought provoking, in spite of some remaining challenges towards broad adoption. These challenges should be faced openly by leveraging the cross-domain expertise and collaboration which has moved the field forward. This includes efforts towards harmonization. No matter which of the 3D, 2D, or 1D MRI approaches are chosen, current MRI protocols used for renal size assessment lack standardization and recognized normal values. To close this gap, it is essential to obtain normal reference values for kidney size and kidney size changes that are normalized to age, sex, and body mass index, based on harmonized MRI protocols. Such efforts will provide consensus-based technical recommendations for clinical translation of renal size assessment with MRI. It is very timely that such activity is initiated and spearheaded by the MRI community and related expert groups. This initiative should not be limited to experimental and clinical studies, but should also take advantage of the rich repositories of data obtained from large-scale population MRI studies, like the UK Biobank or the German National Cohort [42, 65, 128]. These datasets are treasure troves with abundant potential to train ML algorithms to help bring kidney size assessment into routine clinical practice. Leveraging this potential would also help us to decipher the relationships between characteristic signatures obtained from kidney size assessment and molecular profiles, biochemical markers, medical history, clinical correlates, and lifestyle data included in large cohort studies. These insights would further our understanding of the associations and determinants of renal disease. They would also promote extension of the FDA and EMA guidelines on the use of kidney size assessment in support of standardization and reproducibility. This would benefit physiology, nephrology, and related fields, involving clinicians and patients, and provide a springboard for new insights into renal physiology and pathology.
